# Salvage surgery in head and neck cancer

**DOI:** 10.1111/odi.13582

**Published:** 2020-09-21

**Authors:** Stijn van Weert, C. René Leemans

**Affiliations:** ^1^ Department of Otolaryngology‐ Head and Neck Surgery Amsterdam University Medical Centers Amsterdam The Netherlands

**Keywords:** head and neck cancer, organ preservation, prognosticators, salvage surgery

## Abstract

Salvage surgery after failed organ preservation treatment offers challenges for both the patient and the surgeon. The outcome is often uncertain and even today, 5‐year overall survival does not exceed 50 per cent. The chemoradiotherapy induced toxicity asks for meticulous discussion and planning in a multidisciplinary manner in a changing environment of increasing incidence of human papillomavirus induced oropharyngeal tumours, evolving surgical techniques and patient participation. Herein, we discuss the latest literature on salvage surgery and the need for identifying the proper prognosticators to ensure for an optimal treatment plan in potentially salvageable patients.

## 1 INTRODUCTION

Salvage surgery in Head and Neck Cancer (HNC) after failed (chemo)radiotherapy is a complex and increasingly important issue with high stakes for the patients. Patients eligible for SS have previously been through the process of HNC treatment with accompanying anxiety and uncertainties. Therefore, they should be guided accordingly and informed in a truthful and concise manner meaning that salvage surgery is a last resort treatment with an often uncertain outcome both considering cure and function impacting quality of life. Salvage surgery should never be considered a fall back option in case patients elect organ preservation treatment over an advised primary surgical treatment. Performing surgery in previously irradiated tissue, especially when combined with systemic treatment enhancing toxicity, is a very difficult and comes with many both short‐ and long‐term complications.

Although surgical and radiation techniques have improved, salvage surgery remains a journey not easily embarked on with current success rates often not exceeding 30%.

This paper sets out to give an overview of current literature with regard to prognosticators in salvage surgery in light of developments such as increasing incidence of human papillomavirus in oropharyngeal squamous cell carcinoma, the use of transoral robotic surgery, necessity of multidisciplinary management and the increasing awareness for value‐based health care.

## 2 EVOLVEMENT OF SALVAGE SURGERY

Although salvage surgery has always played a role in HNC, its prospects have changed over the last decades with the introduction of combined modality treatment for advanced stage disease. The use of mainly cisplatin and later cetuximab in platinum unfit patients has added toxicity causing bigger challenges for uneventful outcome in salvage surgery (Bonner et al., 2006; Pignon, Bourhis, Domenge, & Designé, 2000; Rovira et al., 2017). Every head and neck surgeon has experienced the setbacks of poor healing tendency and disappointing functional outcome caused by severe fibrosis and inferior perfusion, despite reports of improved outcome over the last two decades (Jayaram et al., 2016). The use of transoral robotic surgery (TORS) should currently be added to the surgeon's armamentarium. TORS can be employed in salvage surgery for mainly early‐stage recurrent disease and can be utilized for salvage in well‐selected cases (White et al., 2013).

## 3 PROGNOSTICATORS IN SALVAGE SURGERY

### 3.1 Site

**Figure 1:**
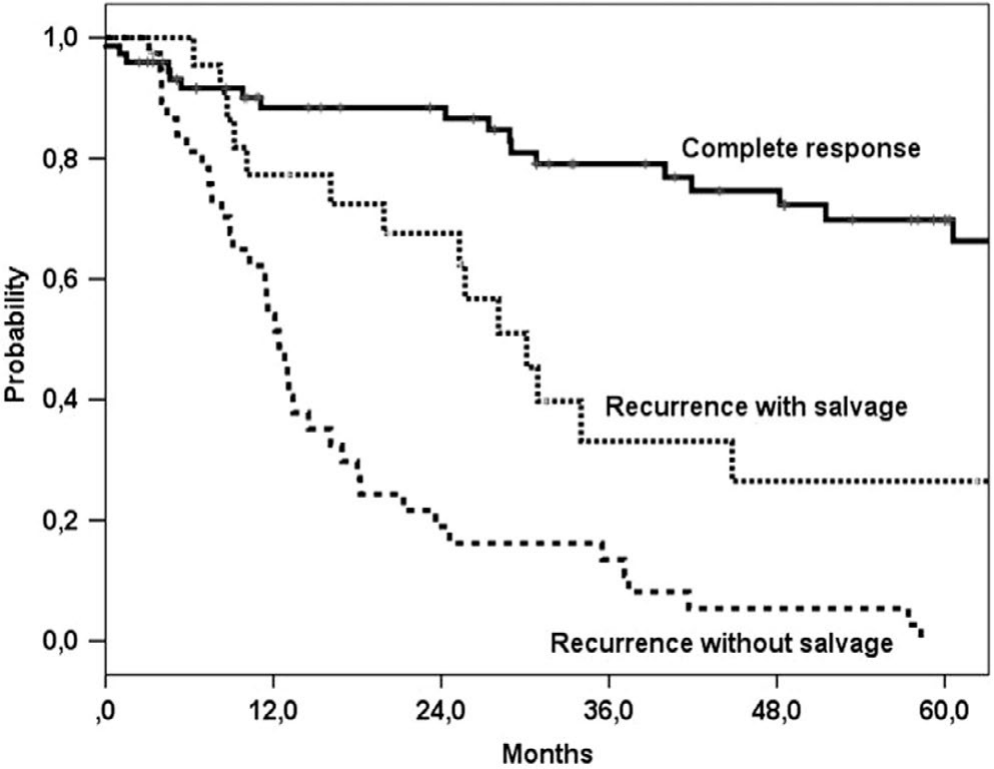
Overall survival for laryngeal and hypopharyngeal cancer with initial chemoradiation treatment with and without salvage surgery. Note the 5‐year overall survival rate after salvage of 27% (van der Putten et al., 2015). (Reprinted with permission)

Success rates in salvage surgery differ per site. Historically, reported outcome in recurrent laryngeal cancer is relatively good, specifically in early‐stage recurrences (Bonner et al., 2006; Chung et al., 2019; Chung, Park, Kwon, & Rho, 2020; Elbers et al., 2019; Goodwin, 2000; van der Putten et al., 2015). Laryngeal recurrences are relatively salvageable due to the possibilities of achieving adequate surgical margins and low nodal spread in early‐stage recurrences. Goodwin (2020) reported a 2‐year overall survival (OS) of well above 60% (83.4% for recurrent stage I/II). Since salvage pharyngo‐laryngectomy is the mainstay of treatment in recurrent hypopharyngeal squamous cell carcinoma (SCC), these series are often analysed together with laryngeal recurrences. Outcome in recurrent hypopharyngeal SCC is inferior to recurrent laryngeal SCC. Complication rate is higher in salvage PL probably due to high percentage of prior chemotherapy and the notoriously poor outcome of hypopharynx cancer (Chung et al., 2019; Elbers et al., 2019; van der Putten et al., 2015). Van der Putten et al. (2015) found a 5‐year OS of 27% for salvage (pharyngo)laryngectomy after primary chemoradiation (Figure 1).

On the other side of the spectrum is neck recurrence. For an isolated neck recurrence, OS drops to below 20% at 18 months (Chung et al., 2020). Radicality in salvage neck dissection is often difficult to achieve, specifically in case of extracapsular spread amidst of fibrosis with limited or no options for re‐irradiation. Adjuvant chemoradiation in the salvage setting is seldom possible because of additional induction of non‐acceptable toxicity.

Oral cavity squamous cell carcinoma (OCSCC) is different in that respect since surgery (with or without radiotherapy) is the primary treatment option. Reported recurrence rates are 25%–45% and even 50% for advanced stage disease. Locoregional recurrence after salvage surgery is around 60% (Lim, Lim, Kim, Byeon, & Choi, 2010; Tam et al., 2017). Several reports have shown that presence of lymph node metastasis at time of SS and positive surgical margins are negative prognosticators (Ho et al., 2014; Matsuura et al., 2018). In case of positive surgical margins, (chemo)re‐irradiation does not seem to improve OS as reported by Zenga et al. (2019) in a multi‐institutional study of both OCSCC and oropharyngeal squamous cell carcinoma (OPSCC) after initial surgery with or without radiotherapy.

Oropharyngeal squamous cell carcinomas can both be treated surgically (early stage) and with primary (chemo)radiation. Due to the increasing incidence of HPV‐positive OPSCC, this particular subsite has been highlighted since HPV‐positive OPSCC is a biologically different disease (Ang et al., 2010). Although HPV‐positive tumours generally have a more favourable outcome, still more than 10% of patients experience (loco)regional failure. HPV‐positive OPSCCs do have a superior outcome in salvage surgery (Fakhry et al., 2014). The addition of TORS in the salvage setting (if feasible in very selected cases) seems to be advantageous compared to open surgical approaches with regard to functional outcome and complication rate (White et al., 2013). Table 1 gives an overview of reported 2‐ and 5‐year OS for OCSCC and OPSCC (Agra et al., 2006; Chung et al., 2020; Hay et al., 2019; Horn et al., 2016; Liu et al., 2007; Nichols et al., 2011; Philouze et al., 2017; Quinlan‐ Davidson et al., 2017; Righini et al., 2012; Sun, Tang, Yang, & Hu, 2009; Tam et al., 2017; Zafereo et al., 2009; Zenga et al., 2019). In general, a non‐laryngeal recurrence is considered a relative negative prognosticator.

**Table 1:** Reported 2‐ and 5‐year overall survival (OS) after salvage surgery for oral cavity (OC) and oropharyngeal (OP) squamous cell carcinoma

Author	*N*	Site	2‐year OS (%)	5‐year OS (%)
Liu et al. (2007)	1,282	OC		31.6
Tam et al. (2017)	293	OC		43
Quinlan‐Davidson et al. (2017)	78	OC		59
Sun et al. (2009)	81	OC		20
Chung et al. (2019)	73	OC		54.8
Horn et al. (2020)	32	OC		41.7
Zafereo et al. (2009)	41	OP	34	28
Nichols et al. (2011)	32	OP	64	43
Righini et al. (2012)	105	OP	31	21
Philouze et al. (2017)	52	OP	43	31
Hay et al. (2019)	25	OP		44
Agra et al. (2006)	264	OC/OP		32.3
Zenga et al. (2019)	102	OC/OP		31

### 3.2 Stage

In his report in 2000, Goodwin (Bonner et al., 2006) stated that tumour stage is a stronger prognosticator for salvage outcome than site. Indeed, advanced stage HNC has a higher recurrence rate and warrants primary chemoradiation or extensive primary surgery with or without chemoradiation. In his prospective study, Goodwin (2000) found a 2‐year disease‐free survival postsalvage surgery of 73%, 67%, 33% and 22% for stage I, II, III and IV, respectively (*p* = .0005). Therefore, he concluded that recurrent stage was a highly significant predictor of recurrence‐free survival where he could not confirm this for specific sites. The fact that stage means more than site has been supported by the majority of authors, with the important note that that data on non‐laryngeal advanced stage disease are sparse (Elbers et al., 2019; Hamoir et al., 2018; Pivot et al., 2001; van der Putten et al., 2015).

### 3.3 Organ preservation strategies: Influence of chemotherapy

Since the emergence of combined modality treatment, advanced stage HNC aimed at organ preservation is treated with radiotherapy combined with cisplatin in a concurrent fashion. Induction chemotherapy, for example, docetaxel, fluorouracil plus cisplatin (TPF), may be used to assess chemosensitivity and/or to reduce the radiation field. It is known that the addition of platinum‐based therapy to radiation gives a survival benefit of 4%–8%. Besides this positive effect, chemotherapy also increases toxicity making SS more challenging. It has been reported that previous chemotherapy in salvage candidates for HNC is negative prognosticator. The primary choice for chemoradiation portends an aggressive course of the disease‐advanced stage disease or high grade features—which could be predictive of a recurrence (Gillison et al., 2019). As for cisplatin used in the re‐irradiation setting after SS, improved disease‐free survival is reported without improvement of OS (Janot et al., 2008).

### 3.4 Human papillomavirus

The incidence of Human Papillomavirus (HPV)‐positive OPSCCs is increasing. A new staging system for p16‐positive OPSCC has been introduced in the eight edition of the UICC/AJCC (Brierley, Gospodarowicz, & Wittekind, 2017). Since its behaviour is distinct, efforts are made to tailor primary treatment. This mostly concerns de‐escalation of treatment for which the first trials have reported results regarding platinum‐based superiority over cetuximab in combined modality treatment (Mehanna et al., 2019; Gillison et al., 2019). All these effort may influence outcome in the salvage surgery setting. De‐escalation trials regarding adjuvant treatment are expected to report results over the next few years (Howard et al., 2018; Owadally et al., 2015). Ma et al. (2020) have presented preliminary results with regard to de‐escalation of cumulative radiotherapy dose in the postoperative chemoradiation setting. Latter authors reported a 2‐year local control rate of 96.2% and 98.7% 2‐year OS with no reported post‐RT toxicity >grade 3. These results indicate that de‐escalation in HPV‐positive OPSCCs seems feasible although longer follow‐up is warranted. Fakhry et al. (2014) already reported on better outcome in salvage surgery for HPV‐positive OPSCCs. Firstly, recurrence‐free survival is longer in HPV‐positive tumours. Secondly, OS in patients with disease progression after locoregional failure was superior in HPV + cases (*p* < .001) and in HPV + patients who underwent salvage surgery (*p* = .004) (Fakhry et al., 2014).

Early‐stage HPV + OPSCCs are preferably primarily treated surgically to attempt to avoid radiotherapy‐induced toxicity. In case of clear margins of the index tumour and limited nodal involvement (single node without extracapsular spread), surveillance is sufficient. In this group of relatively young patients, toxicity reduction is key to prevent xerostomia, dysphagia, carotid artery atherosclerosis and risk reduction for a radiotherapy‐induced tumour. The introduction of TORS has improved the accessibility for oropharyngeal resection and has also taken its role in the unknown primary setting by means of tongue base mucosectomy increasing the identification rate from 40% to 80% (van Weert et al., 2020). Because of possible primary avoidance of toxicity, HPV + OPSCC patients can benefit from (adjuvant)(chemo)radiation in case of locoregional failure.

### 3.5 Margins and N‐status

In recurrent laryngeal cancer, surgical margins are relatively easily achieved. In general, however, recurrences in HNCs are often poorly delineated with submucosal growth (Goodwin, 2000; Ho et al., 2014; Zenga et al., 2019). Proper microscopic margin assessment in previously irradiated tissue is challenging. Margin assessment of this tissue often leads to disappointing histopathological results with little back up treatment options, which is especially the case in patients eligible for salvage surgery after previous chemoradiation for advanced stage disease (Zenga et al., 2019; Hamoir et al., 2018). Positive margins have been reported in over 20% of salvage cases (Zenga et al., 2019). Multiple studies have shown that a clear margin is an independent positive predictor for survival (Matsuura et al., 2018; Nichols et al., 2011; Zafereo et al., 2009).

Neck recurrence is correlated with poor outcome in salvage surgery, both isolated and in combination with a local recurrence. The most favourable outcome of salvage neck dissection is reported in patients with an initial treatment with surgery alone and a N1 recurrence, preferably in the undissected neck (Lim et al., 2010). Extracapsular spread is a well‐established risk factor for recurrence for HNC although a study by Lewis et al. could not confirm this for OPSCC (Lewis, Carpenter, Thorstad, Zhang, & Haughey, 2011). Although treatment regimens for HPV + and HPV− patients are currently equal with regard to adjuvant treatment for the neck (RT in case of > N1; CRT in case of extracapsular spread), recent studies have suggested that adjuvant radiotherapy alone for extracapsular spread in HPV + cases may suffice and that extracapsular spread should be reported in grades (1–4) as suggested by Sinha et al. (An et al., 2017; Sinha, Lewis, Piccirillo, Kallogjeri, & Haughey, 2012). Eventual neck dissection in case of recurrent node(s) with extracapsular spread may lead to vessel and nerve sacrifice due to the extracapsular spread combined with severe fibrosis. Adequate margins will often be difficult to achieve and may lead to a disappointing outcome.

### 3.6 Impact of disease‐free interval

The time interval between initial treatment and recurrence (disease‐free interval) is impacting outcome of salvage surgery. The majority of recurrent HNCs are diagnosed within 18 months after initial treatment (Hamoir et al., 2018; Stell, 1991). A short DFI is a poor prognosticator. Some authors use a cut‐off point of 6 months (because of definition of persistent versus recurrent disease) and found significantly different OS rates where others use 12 months (Hamoir et al., 2018; Liao et al., 2008; Lim et al., 2010; Stell, 1991). Stell (1991) reported a 20% drop in OS in case of a DFI <9 months. A short DFI may reflect aggressive disease with limited response to treatment and problematic salvage scenarios (Ho et al., 2014).

### 3.7 The role of the multidisciplinary team

Over the last decades, designated HNC centres have been formally recognized in many Western countries. This recognition of HNC centres is in line with the general accepted advantage of centralization of low volume and high complex care. Within these centres, multidisciplinary teams (MDTs) have been established to provide optimal care, including the framework of salvage surgery. Each HNC patient is discussed for a tailor made treatment plan. To objectify the added value of MDT meetings, several studies have analysed its role. Results show that MDT discussed cases, mainly stage IV patients, have superior outcome with regard to 5‐year OS. MDT discussed patients were more likely to receive multi‐modality treatment than non‐MDT discussed patients (Friedland et al., 2011; Liao et al., 2016; Philouze et al., 2017; Pignon et al., 2000). Patient referral to a tertiary centre with MDT has reportedly led to changes in staging and treatment in up to 60% of cases (Bergamini et al., 2016). In the recurrent/ salvage setting, there is still room for improvement. Guy et al. (2019) reported a high number of uniquely discussed cases suggesting that recurrent cases are not routinely discussed. In some cases, surgery had been performed prior to the MDT meeting (Guy et al., 2019). Overall, the presence of experts in the field in a MDT leads to implication of current and novel evidence‐based treatments, reduced time to treatment and enrolment in clinical trials for patients in the metastatic and recurrent setting not eligible for curative treatment. Attention for attributing factors as dental and nutritional status is better implemented in a MDT structure (Bergamini et al., 2016; Kelly, Jackson, Hickey, Szallasi, & Bond, 2013). Table 2 summarizes the potential positive prognosticators in SS.

**Table 2:** Summary of positive prognosticators in HNC salvage surgery

Positive prognosticators in Salvage Surgery
Laryngeal recurrence
Early‐stage recurrence
No previous chemotherapy
HPV positivity (OPSCC)
Clear surgical margins
≤N1 and no extracapsular spread
DFI > 6 months
MDT involvement
No comorbidities
Adequate perioperative nutritional/ electrolyte status

Abbreviations: DFI, disease‐free interval; MDT, multidisciplinary team; OPSCC, oropharyngeal squamous cell carcinoma.

## 4 PATIENT SELECTION AND OPTIMIZATION

In addition to tumour characteristics, adequate patient selection and optimization in the pre‐, per and postoperative period is paramount. Patients should have a realistic perspective of cure and preservation of vital functions such as swallowing and speech. Patients with poor functional status after primary treatment are likely to experience further deterioration. These patients need to be able to undergo extensive surgery with often the use of free flaps necessitating the need for proper perfusion. Patients with a medical history of poorly controlled diabetes or cardiovascular disease are therefore at risk for a complicated postoperative course. Comorbidities have proven to be an important factor to consider (Kim et al., 2015). Specifically in case of salvage surgery for toxicity‐induced indications such as recurrent pneumonia, aspiration and cartilage necrosis in patients treated for advanced laryngeal or hypopharyngeal cancer, chances of a complicated course are realistic. Several authors have suggested to use a prediction model for adequate patient selection. Use of the Charlson Age Comorbidity Index (CACI) has been advocated (Hamoir, Holvoet, Ambroise, Lengelé, & Schmitz, 2017; Kim et al., 2015).

In the present time of increasing numbers of HPV‐positive OPSCC patients, there is a distinct group of young and relatively healthy patients eligible for salvage surgery even by means of TORS in select cases, thus avoiding open surgical approaches. White et al. (2013) described the advantages of TORS over open surgery with fewer complications on both short as long term. On the other hand, this group represents a small part of potentially salvageable HNC patients and a substantial number of recurrent OPSCC is still HPV‐negative and of older age with accompanying comorbidities (Zafereo et al., 2009).

The “ideal candidate” for salvage surgery could be defined as a young patient having an early‐stage local laryngeal recurrence without any comorbidities. In reality, this category of patients makes up for a very modest percentage of salvage candidates. Patient selection should be done after extensive and realistic consultation with the patient and his family and to ensure for modern‐day shared decision‐making. MDT discussion of salvage candidates contributes to proper decision‐making, specifically in case of a realistic chance of adjuvant treatment.

## 5 RECONSTRUCTION IN SALVAGE SURGERY

To optimize the chances of uneventful recovery, introduction of well‐vascularized tissue in a toxicity exposed area is vital. Initially, pedicled flaps were used for this purpose such as the pectoralis major flap and the latissimus dorsi flap (Ariyan, 1979). These versatile flaps have an excellent reliability and can be used for multiple purposes like pharyngeal closure (myocutaneous) after pharyngo‐laryngectomy and for improving healing tendency and prevention of complications (e.g., pharyngocutaneous fistula). Even today, these flaps are very useful in select cases. Due to the limited geometry and bulkiness of these flaps, however, the introduction of free flaps has shown to be a major improvement. Introduction of healthy and well‐perfused tissue into a previously treated area allows not only for better healing, but more so for better functionality. The pliability of, for example, the free radial forearm flap (FRFF) or the anterolateral thigh flap (ALTF) after hemi‐ or major glossectomy has contributed immensely to speech and swallowing rehabilitation. Prerequisites for successful microvascular surgery are an experienced surgical team as well as proper equipment and preparation. The team should be able to deal with a vessel depleted neck and should be skilled in using grafts to increase pedicle length if needed. In case of using a FRFF, ALTF or a composite flap like the fibula flap, pedicle length is usually sufficient to reach the contralateral neck in case the previously treated neck is not suited for anastomosis. This can either be due to poor quality vessels as a result of prior (chemo)radiation or vessel sacrifice (Hanasono, Barnea, & Skoracki, 2009; Jacobson, Eloy, Park, Roman, & Genden, 2008; Mulholland et al., 1993). Flaps with a relatively short pedicle as the scapula flap may warrant the use of grafts. Additional skills in recognizing flap perfusion failure and performing instant flap salvage are mandatory.

## 6 COMPLICATIONS AFTER SALVAGE SURGERY

Since salvage is performed in previously treated tissue, complication rates are relatively high. Previously induced fibrosis and toxicity deteriorate healing tendency. Goodwin (Bonner et al., 2006) reported complication rates of 6% and 30% for early and advanced stage recurrences, respectively. Complication rates as high as 67% have been reported with additional risk in patients previously treated with chemoradiation or needing neck dissection. Besides perioperative complications, long‐term complications as progressive fibrosis, prolonged feeding tube or tracheotomy dependence are not rare after salvage surgery (Kostrzewa et al., 2010; Nichols et al., 2011; White et al., 2013; Zafereo et al., 2009). The Clavien–Dindo classification has been adopted for head and neck surgery to ensure uniformity and reproducibility of complication registration (Dindo, Demartines, & Clavien, 2004). A recent study by Philips et al. showed that medical complications have a significant impact on survival in SS rather than surgical complications. Hypothyroidism and liver disease played a predominant role in this case study. These results demonstrate the importance of multidisciplinary care and tailored treatment (Philips et al., 2019).

## 7 CONCLUSION

Improvements have been made over the last decades in HNC treatment as for salvage. Introduction of designated head and neck centres, increasing use of MDTs, evolution in reconstructive surgery and improved patients optimization have all contributed. The increasing number of HPV‐positive OPSCC patients may play a role in improved outcome in SS. Jayaram et al. (2016) described this improvement over the past two decades for OPSCC specifically with a 5‐year OS of 18% in the pre‐2000 era amounting to 50% in the present time. The ongoing sophistication in TORS and its progressive use has led to less open surgical approaches for OPSCC recurrences. The upcoming interest for value‐based health care and shared decision‐making has contributed to patient participation (Roman, Awad, & Patel, 2015). Patients are consulted accordingly in case of presumed salvage surgery. Patients not eligible for salvage surgery should be considered for participation in immunotherapy clinical trials (Gavrielatou, Doumas, Economopoulou, Foukas, & Psyrri, 2020). Although major improvements have been made in managing patients with recurrent and residual HNC, a 5‐year postsalvage OS ranging from 30% to 50% nowadays is still modest. Time should be taken to extensively discuss a case both in a MDT as with the patient. Every possible prognosticator should be carefully weighed (Leemans, 2017). In case of poor general condition, short DFI, advanced stage disease and significant nodal tumour burden, a plan for salvage surgery should at least be reconsidered. The patient should be informed thoroughly so to decide whether these high stakes of salvage surgery are worth the effort and uncertainty. SS is indeed a last resort treatment with an often unpredictable outcome and will remain so in the near future. Further development of predictive modelling may aid in decision‐making in salvage surgery.

## CONFLICT OF INTEREST

None to declare.

## AUTHOR CONTRIBUTIONS

**Stijn van Weert:** Conceptualization; Data curation; Formal analysis; Methodology; Writing‐review & editing. **C. René Leemans:** Conceptualization; Formal analysis; Writing‐original draft; Writing‐review & editing.

### Peer Review

The peer review history for this article is available at https://publons.com/publon/10.1111/odi.13582.
